# Young bone marrow transplantation preserves learning and memory in old mice

**DOI:** 10.1038/s42003-019-0298-5

**Published:** 2019-02-20

**Authors:** Melanie M. Das, Marlesa Godoy, Shuang Chen, V. Alexandra Moser, Pablo Avalos, Kristina M. Roxas, Ivy Dang, Alberto Yáñez, Wenxuan Zhang, Catherine Bresee, Moshe Arditi, George Y. Liu, Clive N. Svendsen, Helen S. Goodridge

**Affiliations:** 1 Board of Governors Regenerative Medicine Institute, Cedars-Sinai Medical Center, 8700 Beverly Boulevard, Los Angeles, CA 90048 USA; 2 Department of Biomedical Sciences, Cedars-Sinai Medical Center, 8700 Beverly Boulevard, Los Angeles, CA 90048 USA; 3 Department of Pediatrics, Cedars-Sinai Medical Center, 8700 Beverly Boulevard, Los Angeles, CA 90048 USA; 4 Biostatistics and Bioinformatics Research Institute, Cedars-Sinai Medical Center, 8700 Beverly Boulevard, Los Angeles, CA 90048 USA

## Abstract

Restoration of cognitive function in old mice by transfer of blood or plasma from young mice has been attributed to reduced C–C motif chemokine ligand 11 (CCL11) and β2-microglobulin, which are thought to suppress neurogenesis in the aging brain. However, the specific role of the hematopoietic system in this rejuvenation has not been defined and the importance of neurogenesis in old mice is unclear. Here we report that transplantation of young bone marrow to rejuvenate the hematopoietic system preserved cognitive function in old recipient mice, despite irradiation-induced suppression of neurogenesis, and without reducing β2-microglobulin. Instead, young bone marrow transplantation preserved synaptic connections and reduced microglial activation in the hippocampus. Circulating CCL11 levels were lower in young bone marrow recipients, and CCL11 administration in young mice had the opposite effect, reducing synapses and increasing microglial activation. In conclusion, young blood or bone marrow may represent a future therapeutic strategy for neurodegenerative disease.

## Abstract (web summary)

Melanie Das et al. demonstrate that transplantation of young bone marrow preserves the cognitive function of old recipient mice. This study suggests that microglial rejuvenation via peripheral manipulation of the hematopoietic system may be sufficient to delay a cognitive decline during aging.

## Introduction

Surgically attaching old mice to young mice so that they share a circulatory system (heterochronic parabiosis) has been reported to rejuvenate old mice and accelerate aging in young mice (reviewed in refs. ^1,2^). Rejuvenation of the brain, heart, liver and pancreas of old parabionts by young blood is thought to be partly due to effects on stem cell populations (reviewed in refs. ^1,2^). In particular, improved cognitive function has been attributed to increased neurogenesis^3,4^ and synaptic plasticity^5^, as well as better brain vascularization^4^ and myelination^6^. A single blood exchange between old and young mice, which replaces the blood without organ sharing or complications associated with the parabiosis procedure, has also recently been reported to have similar effects^7^.

The blood contains multiple components that influence tissue/organ function and could therefore be responsible for aging/rejuvenation in parabiotic mice, including hematopoietic cells, as well as soluble factors. Plasma transfer experiments have suggested that changes in soluble factors in the circulation are responsible for brain rejuvenation in old mice joined to young mice^5,8^. Soluble factors of both non-hematopoietic and hematopoietic origin likely contribute to the observed effects. For instance, restoration of the regenerative potential of skeletal muscle in old mice joined to young mice has been attributed to elevated testosterone levels^9^. Changes in circulating levels of inflammatory cytokines and chemokines may also underlie some of the observed aging/rejuvenation effects of parabiosis. In particular, several chemokines have been reported to be elevated in the circulation of old mice and in young mice joined to old mice^3^.

Circulating levels of the C–C motif chemokine ligand 11 (CCL11, also known as eotaxin-1) and β2-microglobulin have previously been reported to increase with age in mice and humans, and shown to promote brain aging when administered to young mice^3,10,11^. Both CCL11 and β2-microglobulin can be produced by a diverse range of cell types, and the tissue(s)/organ(s) responsible for their elevated levels during aging have not been defined. Thus, the role of the hematopoietic system in these effects is unclear.

CCL11 and β2-microglobulin are thought to act by suppressing neurogenesis in the hippocampus, because neurogenesis was enhanced in old mice rejuvenated by parabiosis or plasma transfer, and injection of CCL11 or β2-microglobulin into young mice suppressed neurogenesis^3,10^. However, neurogenesis in the rejuvenated old mice was only partially restored compared to young mice, and the role of neurogenesis in the adult brain is controversial, with some studies suggesting that it is of minimal importance for maintenance of hippocampal function^12–14^. Thus other mechanisms may be responsible for the rejuvenated cognitive function in old mice undergoing heterochronic parabiosis or plasma transfer. Indeed, while stem cell populations in the neurogenic niche have been closely examined, it is not known whether aging-associated changes in glial cells are also reversed.

We therefore established a heterochronic bone marrow transplant (BMT) model to determine the specific influence of systemic hematopoietic aging on cognitive function, including glial cells in the hippocampus. This approach also allowed us to evaluate the long-term beneficial impact of a young hematopoietic system on the aging brain, and define the role of the hematopoietic system in aging-associated elevation of circulating levels of CCL11 and β2-microglobulin. Irradiation (9 Gy, split dose) delivered without head shielding prior to injection of donor bone marrow cells enabled us to exclude the impact of neurogenesis, because irradiation is known to inhibit the proliferation of neural progenitors^15,16^.

We found that reconstitution of old mice with young, but not old, hematopoietic cells prevented cognitive decline. BMT achieved preservation of cognitive function for at least 6 months, despite suppression of neurogenesis. Instead, microglial activation was reduced, and synaptic connections were maintained. Our data also attribute the aging-associated elevation of circulating β2-microglobulin levels to non-hematopoietic cells. In contrast, the increased CCL11 appears either to be of hematopoietic origin or to be produced by non-hematopoietic cells under hematopoietic control, and our data implicate CCL11 in aging-associated microglial activation and synaptic loss.

## Results

### Impact of heterochronic BMT on cognitive function

**Fig. 1:**
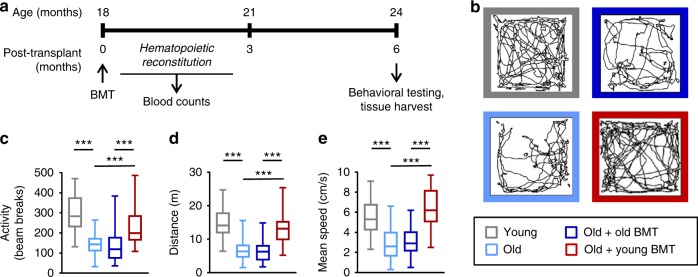
Transplantation with young bone marrow preserved activity in old mice. **a** Timeline of bone marrow transplantation (BMT), hematopoietic reconstitution (0–3 months post-transplantation), behavioral analysis and tissue harvest for histology and molecular analyses (6 months post-transplantation). Old (18-month) mice received bone marrow from either young (4-month) or old (18-month) donors, and cognitive function was evaluated 6 months later. Non-transplanted old (24-month) and young (4-month) control mice were also evaluated. **b**–**e** Activity (**b**, **c**; includes horizontal locomotion and vertical rearing), distance covered (**d**), and speed (**e**) were assessed during four 5-min intervals over a 20-min period in an open field test, and the mean activity, distance and speed were calculated for each mouse. *n* = 32–51 mice per group, pooled from 3 independent experiments. Justification for pooling was confirmed by multi-ANOVA analysis. Similarly statistically significant differences were observed in all independent experiments. Box and whisker plots show median, 25th and 75th percentile, maximum and minimum values. ***p < 0.001 (ANOVA with Tukey-Kramer post-hoc test)

To evaluate the impact of hematopoietic age on cognitive function, we established a model of heterochronic BMT (Fig. 1a). Old (18-month) recipient mice were irradiated (without head-shielding) prior to injection of donor bone marrow cells from either young (4-month) or old (18-month) mice to achieve nearly complete (>90%) reconstitution with donor bone marrow (Supplementary Fig. 1a). Lymphocyte counts were initially slightly lower in old bone marrow recipients than young bone marrow recipients, but similar by 3 months post-transplantation (Supplementary Fig. 1b).

Behavioral testing was performed 6 months post-transplantation to assess activity and cognition (Fig. 1a). In an open field test, old control mice exploring a novel environment were less active than young control mice during the first 20 min of evaluation (Fig. 1b–e). Remarkably, however, young bone marrow recipients, but not old bone marrow recipients, were more active than old control mice (Fig. 1b–e). At the end of a 1-h assessment period, however, both transplanted groups and old control mice were similarly active (Supplementary Fig. 2a–c), suggesting that general wellness does not underlie the differences in exploratory behavior observed during the first 20 min of testing.

**Fig. 2:**
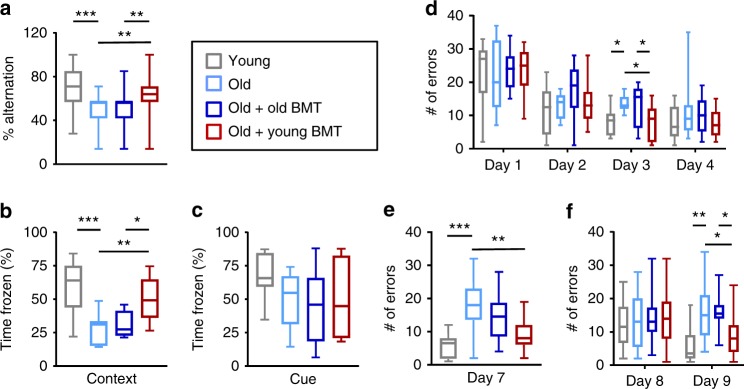
Transplantation with young bone marrow prevented cognitive decline in old mice. **a** Spontaneous alternation between the arms of a Y-maze was assessed over an 8-min period. *n* = 44–46 mice per group, pooled from 3 independent experiments. Justification for pooling was confirmed by multi-ANOVA analysis. Similarly statistically significant differences or trends towards significance were observed in all independent experiments. **b**, **c** Memory of a prior electric shock was evaluated upon re-exposure to the same environment (context-dependent fear conditioning; **b**) or a tone that had preceded the shock (cue-dependent fear conditioning; **c**) by assessing freezing during a 5-min re-exposure period. *n* = 11 mice per group. **d**–**f** In a Barnes Maze test, the ability of mice to discover and then recall the location of an escape hole was evaluated during the learning phase (days 1–4; **d**), after a 2-day break (day 7; **e**), and following re-positioning of the escape hole (days 8–9; **f**). The number of errors made prior to successful location of the escape hole was recorded. *n* = 10–15 mice per group. Box and whisker plots show median, 25th and 75th percentile, maximum and minimum values. *p < 0.05, **p < 0.01, ***p < 0.001 (ANOVA with Tukey-Kramer post-hoc test)

Exploratory behavior is thought to originate in the hippocampus^17^. We therefore evaluated hippocampus-dependent learning and memory. In the spontaneous alternation maze (Y-maze), old control mice performed worse than young control mice, but young bone marrow recipients completed more spontaneous alternations between the arms of the maze than both old bone marrow recipients and old control mice (Fig. 2a). This suggests that young BMT preserves spatial and working memory in old mice. The old control mice made fewer total arm entries than the young control mice, and transplantation with neither young nor old bone marrow reversed this (Supplementary Fig. 2d).

A context-specific fear conditioning test was next used to evaluate hippocampus-dependent memory. When placed in the same environment in which they had previously received an electric shock, old control mice froze for shorter periods than young control mice (consistent with impaired memory), while young (but not old) bone marrow recipients froze for longer periods than old control mice (Fig. 2b). In contrast, a cue-specific fear conditioning test that assesses amygdala-dependent memory revealed no difference between any of the groups when mice were re-exposed to an audible cue that had preceded the electric shock (Fig. 2e). Collectively, these results confirm that a young hematopoietic system slows the aging-associated decline in hippocampal function.

Learning, spatial memory and memory recall were next assessed using a Barnes maze^18^. In comparison with old control mice and old bone marrow recipients, young control mice and young bone marrow recipients made only slightly fewer errors when locating an escape hole during the training phase (days 1–4; Fig. 2d). Following a 2-day break, however, young control mice and young bone marrow recipients made fewer errors than old control mice and old bone marrow recipients (day 7; Fig. 2e). The position of the escape hole was then changed and the number of errors made when discovering the new location was similar across all groups (day 8; Fig. 2f). However, the following day, young control mice and young bone marrow recipients made fewer errors compared to old control mice and old bone marrow recipients (day 9; Fig. 2f). In contrast, the time taken to successfully complete the test was similar across all groups on each day of testing (Supplementary Fig. 2c). Collectively, these data demonstrate that a young hematopoietic system preserves recall ability in old mice.

### Impact of heterochronic BMT on neurons and glial cells

**Fig. 3:**
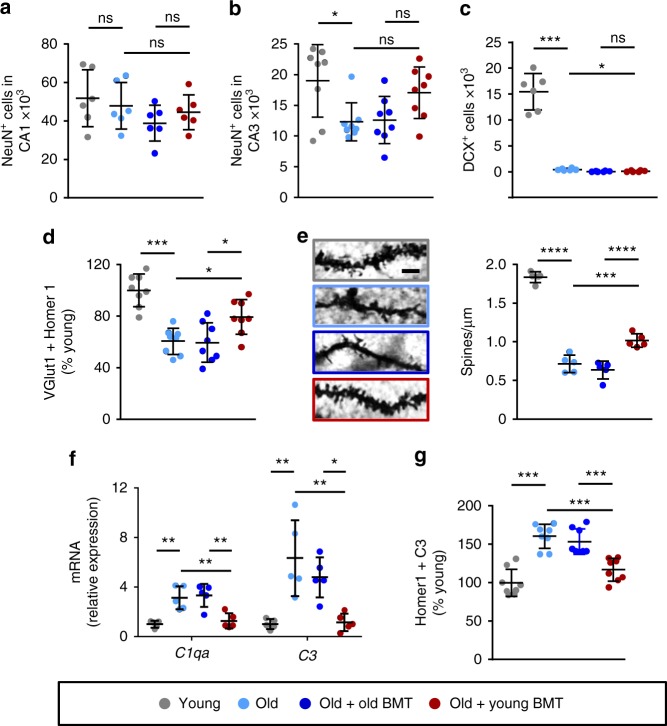
Synapses were preserved in young bone marrow recipients. Neurons and synapses in the hippocampus were evaluated 6 months post-transplantation. **a**, **b** Neuron numbers in the CA1 (**a**) and CA3 (**b**) regions were assessed by NeuN staining. *n* = 6–8 mice per group, pooled from 2 independent experiments. **c** Newly-born neurons were assessed by counting doublecortin (DCX)^+^ cells. *n* = 6 mice per group. **d** Synapses were counted by evaluating co-localization of VGlut1 (pre-synaptic) and Homer1 (post-synaptic). *n* = 8 mice per group, pooled from 2 independent experiments. **e** Spine density was visualized and quantified on Golgi-impregnated slices. Scale bar: 2.5 μm. *n* = 5 mice per group. **f** Complement expression was assessed by RT-PCR (normalized to *Gapdh* mRNA). *n* = 5 mice per group, pooled from 2 independent experiments. **g** C3 deposition on synapses was evaluated by assessing C3 co-localization with Homer1. *n* = 8 mice per group, pooled from 2 independent experiments. The dotplots show mean plus standard deviation. *p < 0.05, **p < 0.01, ***p < 0.001, ****p < 0.0001 (ANOVA with Tukey-Kramer post-hoc test)

We next assessed neuron numbers and synapses in the hippocampus 6 months post-transplantation. Neuron numbers were reduced in the CA3 (but not the CA1) region of the hippocampus in old control mice (Fig. 3a, b and Supplementary Fig. 3a), consistent with previous studies^19^. Following young BMT, we observed a trend towards preservation of neuron numbers in the CA3 region, but it was not statistically significant (Fig. 3b). There was also an aging-associated reduction in the number of doublecortin (DCX)^+^ newly-born neurons in the dentate gyrus, and almost complete ablation of these cells in both young and old bone marrow recipients (Fig. 3c and Supplementary Fig. 3b), presumably due to the irradiation administered prior to transplantation^15,16^. We also assessed 5-bromo-2’-deoxyuridine (BrdU) incorporation in the dentate gyrus of bone marrow recipients at an earlier time point after transplantation (1 month post-transplant, when the mice were 19 months of age). We observed reduced BrdU incorporation in old control mice at this age compared to young control mice, and this was unchanged following transplantation with either old or young bone marrow (Supplementary Fig. 3c–d). Taken together these data indicate that preservation of cognitive function in the BMT model was independent of neurogenesis.

Neuronal loss is normally preceded by synaptic loss in aging and aging-associated diseases^20^, and synaptic plasticity has been reported to improve in old mice following heterochronic parabiosis^5^. Thus, we postulated that synaptic connections may be maintained in young bone marrow recipients. Indeed, we observed an aging-associated decrease in co-localized VGlut1 (pre-synaptic) and Homer1 (post-synaptic) puncta in control mice, and preservation of co-localized puncta in recipients of young, but not old, bone marrow (Fig. 3d and Supplementary Fig. 3e). Moreover, Golgi impregnation of hippocampal neurons revealed a greater spine density in young, but not old, bone marrow recipients compared to old controls (Fig. 3e).

The complement factors C1qA and C3 are both over-expressed in the aging hippocampus and have been implicated in aging-associated cognitive decline^19,21^. C3 deposition on synapses labels them for detection by microglia, thereby inducing synaptic elimination^19,22,23^, which may be aberrantly over-active during aging^20^. We therefore assessed complement expression in the hippocampus, and found that *C1qa* and *C3* mRNA levels were lower in young, but not old, bone marrow recipients (Fig. 3f). We also observed reduced C3 deposition on synapses (co-localized Homer1 and C3 puncta; Supplementary Fig. 3f) in young, but not old, bone marrow recipients compared to old control mice (Fig. 3g).

**Fig. 4:**
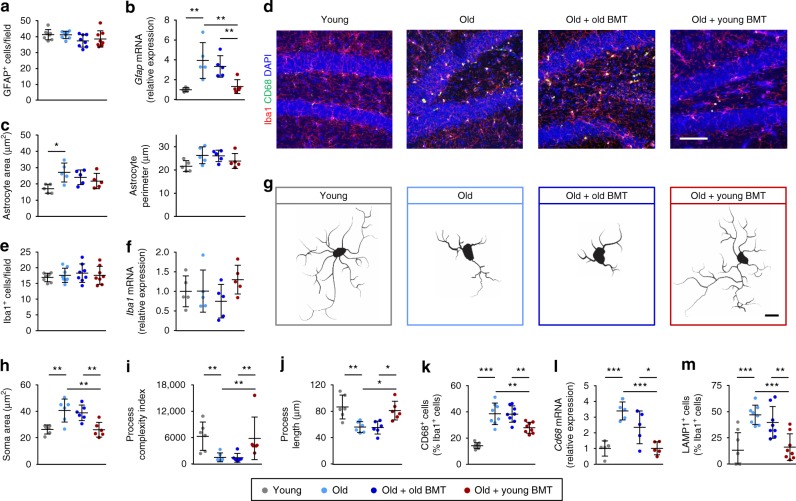
Young BMT reduced the aging-associated activation of microglia. Activation of astrocytes and microglia was evaluated 6 months post-transplantation. **a**, **b** Astrogliosis was evaluated by counting GFAP^+^ cells in the hippocampus (**a**) and by assessing GFAP expression by RT-PCR (**b**). **c** Astrocyte hypertrophy was assessed by measuring the mean area and perimeter of GFAP^+^ cells. **d**–**f** Microglia were quantified by counting Iba1^+^ cells in the hippocampus (**d**, **e**) and by assessing Iba1 expression by RT-PCR (**f**). **g**–**j** The morphology of Iba1^+^ cells (**g**) was assessed by measuring their mean soma area (**h**), process complexity (**i**) and process length (**j**). **k**–**m** Microglial activation was also evaluated by assessing the proportion of Iba1^+^ cells with intense CD68 staining (**k**), *Cd68* mRNA expression by RT-PCR (**l**), and proportion of Iba1^+^ cells with intense LAMP1 staining (**m**). Scale bars: 50 μm in **d**, 5 μm in **g**. RT-PCR data were normalized to *Gapdh* mRNA. For histological analyses, *n* = 5–8 mice per group, pooled from 2 independent experiments; for RT-PCR experiments, *n* = 5 mice per group. The dotplots show mean plus standard deviation. *p < 0.05, **p < 0.01, ***p < 0.001 (ANOVA with Tukey-Kramer post-hoc test)

We next evaluated reactive astrocytes and microglia, which have also been shown to contribute to cognitive decline in aging^23,24^. We found no difference between the groups in the number of reactive glial fibrillary acidic protein (GFAP)^+^ astrocytes in the hippocampus (Fig. 4a and Supplementary Fig. 4a), but higher *Gfap* mRNA expression in old control mice and old bone marrow recipients compared to young controls (Fig. 4b). In contrast, *Gfap* mRNA levels in young bone marrow recipients were reduced to levels seen in young control mice (Fig. 4b). We also evaluated astrocyte hypertrophy by assessing the area and perimeter of GFAP^+^ cells, both of which were increased in old control mice (although only the area reached statistical significance; Fig. 4c). However, we did not observe a statistically significant reduction in astrocyte hypertrophy in the young bone marrow recipients, so preserved cognition could not be attributed to normalization of astrocyte morphology.

Microglia in the hippocampus are specifically vulnerable to aging^25^. While the number of Iba^+^ microglia was similar in all groups (Fig. 4d–f and Supplementary Fig. 4b), their activation state was very different (Fig. 4d, g–m and Supplementary Fig. 4b). Specifically, the microglia of old control mice exhibited a ‘reactive’ morphology^26^, with larger soma, as well as fewer and shorter processes than those of young control mice (Fig. 4g–j). This phenotype was reversed in recipients of young, but not old, bone marrow (Fig. 4g–j). In addition, quantification of reactive microglia by immunostaining for CD68 and LAMP1 revealed that more Iba1^+^ microglia co-stained with these lysosomal markers in old control mice than young control mice (Fig. 4d, k, m and Supplementary Fig. 4b), and *Cd68* mRNA levels were also elevated (Fig. 4l). This phenotype was also completely reversed in recipients of young, but not old, bone marrow (Fig. 4d, k–m and Supplementary Fig. 4b). Thus, synapse preservation in the young bone marrow recipients may be a consequence of reduced phagocytic engulfment of synapses by microglia.

### Impact of heterochronic BMT on CCL11 and β2-microglobulin

We next explored the mechanisms underlying the reduced activation of microglia. Previous studies have shown that donor-derived CD11b^+^ cells can be found in the choroid plexus and meninges of recipient mice following BMT, but rarely infiltrate the brain parenchyma in the absence of acute damage^26^. Similarly, we did not observe donor-derived cells in the brain 3 weeks after transplantation of young GFP-transgenic bone marrow into old mice (Supplementary Fig. 5a), but 3 months post-transplantation, GFP^+^ CD11b^+^ cells were seen in the choroid plexus and meninges (Supplementary Fig. 5a–c). This is consistent with previous reports that choroid plexus and meningeal macrophages are replaced throughout life^26^. However, donor-derived cells were not observed in the brain parenchyma (Supplementary Fig. 5a, d). Thus it is likely that the preservation of cognitive function is not due to direct effects of young hematopoietic cells in the hippocampus.

**Fig. 5:**
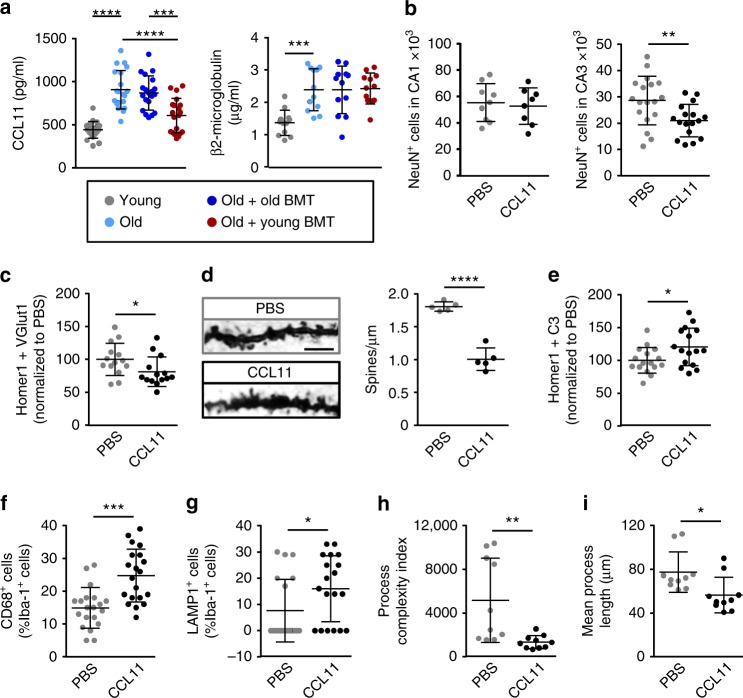
Circulating CCL11 levels were reduced in young bone marrow recipients and CCL11 injection into young mice mimicked hippocampal aging. **a** CCL11 and β2-microglobulin levels in the plasma of control mice and BMT recipients were quantified by ELISA. *n* = 12–20 mice per group, pooled from 2 independent experiments. **b**–**i** CCL11 was administered by intraperitoneal injection into young (4-month) mice (4 injections over 10 days), prior to histological and molecular analyses of the hippocampus. **b** Neuron numbers in the CA1 and CA3 regions were assessed by NeuN staining. *n* = 8–18 mice per group, pooled from 2 independent experiments. **c** Synapses were evaluated by co-localization of VGlut1 (pre-synaptic) and Homer1 (post-synaptic) markers. *n* = 14 mice per group, pooled from 2 independent experiments. **d** Spine density was assessed and quantified by Golgi impregnation of CA3 neurons. Scale bar: 2.5 μm. *n* = 5 mice per group. **e** C3 deposition on synapses was evaluated by C3 co-localization with Homer1. *n* = 16 mice per group, pooled from 2 independent experiments. **f**–**i** Microglial activation was assessed by measuring the percentage of Iba1^+^ cells with cytosolic co-staining of CD68 (**f**) or LAMP1 (**g**), as well as the complexity (**h**) and length (**i**) of microglial processes. *n* = 19–20 mice per group (CD68 and LAMP1 counts) or 10 mice per group (microglial morphology), pooled from 2 independent experiments. The dotplots show mean plus standard deviation. *p < 0.05, **p < 0.01, ***p < 0.001, ****p < 0.0001 (**a**—ANOVA with Tukey-Kramer post-hoc test; **b**–**i**—Student’s *t*-test)

We therefore next investigated whether heterochronic BMT impacts the levels of circulating factors previously implicated in brain aging, focusing on β2-microglobulin and CCL11. Levels of β2-microglobulin and CCL11, which have been shown to suppress neurogenesis upon systemic injection into young mice, are elevated in the circulation and cerebrospinal fluid (CSF) of old mice and humans, and also increased in young parabionts following heterochronic parabiosis^3,10,11^. As expected, we observed higher plasma levels of both β2-microglobulin and CCL11 in old control mice than young control mice (Fig. 5a). Notably, however, plasma levels of CCL11, but not β2-microglobulin, were reduced in recipients of young (but not old) bone marrow (Fig. 5a). This suggests that non-hematopoietic cells are the main source of β2-microglobulin in old mice, while CCL11 is either derived from hematopoietic cells or produced by non-hematopoietic cells under the influence of hematopoietic cells. Moreover, it raises the possibility that reduced CCL11 production may underlie the preservation of cognitive function in old mice following young BMT.

### Effects of CCL11 injection into young mice

Microglia express higher levels of the CCL11 receptors CCR2, CCR3 and CCR5 than other brain cell types (including neurons and astrocytes)^27^. We therefore hypothesized that increased CCL11 production might be responsible for activation of microglia and aberrant synaptic pruning in old mice. To investigate this, we administered recombinant CCL11 to young mice via intraperitoneal injection, which achieves a physiologically relevant increase in circulating CCL11 levels^3^. Consistent with a previous study^3^, this resulted in reduced numbers of DCX^+^ newly-born neurons (Supplementary Fig. 6a). We also observed a reduction in CA3, but not CA1, region hippocampal neurons (Fig. 5b). The number of synapses (co-localized VGlut1 and Homer1 puncta) also decreased following CCL11 injection (Fig. 5c), as did the average spine density of Golgi-impregnated neurons (Fig. 5d). Moreover, quantification of co-localized Homer1 and C3 puncta showed that systemic CCL11 injection increased C3 deposition on synapses (Fig. 5e), which indicated that the reduction in synapses may be a consequence of pruning by microglia. Interestingly, astrocyte activation (GFAP^+^ cell number and *Gfap* mRNA expression) was unaffected by CCL11 administration (Supplementary Fig. 6b, c), and Iba1^+^ microglial numbers were unchanged (Supplementary Fig. 6d). In contrast, microglia in the hippocampus were more reactive, with increased CD68 and LAMP1 expression, as well as fewer and shorter processes (Fig. 5f–i and Supplementary Fig. 6e). Thus CCL11 injection reproduced the synaptic loss and microglial reactivity observed in old mice.

## Discussion

Previous studies using a similar approach (although with sex mismatched mice and no irradiation) have suggested that heterochronic BMT can extend lifespan^28,29^. We have now shown that heterochronic BMT preserves cognitive function in old mice for at least 6 months. Our data suggest that replacement of old peripheral hematopoietic cells with young ones specifically improves hippocampal memory, likely by preventing synapse loss. Although anxiety could impact certain behaviors, there is no indication that differences in anxiety account for the observed effects. Indeed, a previous study reported that differences in anxiety have a minimal impact on performance in the Barnes maze^30^, and in our study, the old mouse groups made a similar number of total arm entries in the Y-maze, suggesting no apparent differences in anxiety.

Taken together, our data support an emerging model of aging-associated cognitive decline that is in part due to aging of the hematopoietic system. We present data suggesting that young blood can improve cognitive function in old mice by reducing activation of microglia and without rejuvenation of the stem cell niche. Previous parabiosis studies have attributed cognitive aging, at least in part, to reduced neurogenesis^3,4,10^. However, the role of neurogenesis in the adult brain is controversial^12–14^, and our BMT data demonstrate that preservation of cognitive function does not require restoration of neurogenesis. Instead, our data suggest that aging-associated cognitive decline is more likely to be a consequence of activation of microglia and synapse loss, perhaps due to overzealous pruning of synapses by microglia^19,20,22,31^.

Importantly, in contrast to studies of neurodegenerative diseases in which donor-derived monocytes more readily cross the severely compromised blood-brain-barrier to phagocytose debris and clear β-amyloid plaques in the brain parenchyma (reviewed in refs. ^32,33^), cognitive preservation in the old but otherwise healthy mice could not be attributed to donor cell infiltration. Instead, consistent with heterochronic plasma transfer studies^5,8^, it appears that soluble factors in the circulation are responsible. Interestingly, consistent with a previous report^3^, our analyses suggest that CCL11 is an important mediator of aging-associated cognitive decline. CCL11 levels are elevated in the circulation of old mice and humans^3,11^, and CCL11 production has also been reported to be elevated in the brains of old mice^34^.

The reduction in circulating CCL11 levels that we observed in young bone marrow recipients indicates that CCL11 is either derived from hematopoietic cells or produced by non-hematopoietic cells under hematopoietic control. Consistent with the latter hypothesis, increased CCL11 production by choroid plexus epithelial cells has previously been attributed to an aging-associated shift in T cell polarization^34^. CCL11 has also been reported to be produced by activated astrocytes^27^. It remains to be seen whether CCL11 produced inside or outside the brain parenchyma has the biggest impact on the hippocampus. Although circulating levels of CCL11 increase with age^3^ and CCL11 has been shown to be capable of crossing the blood-brain-barrier^35^, it is possible that targeting brain-derived CCL11 production may be sufficient to preserve cognitive function.

In ongoing studies we are investigating whether there is a causal link between increased CCL11 and synaptic pruning by microglia, but we hypothesize that CCL11 promotes cognitive decline by directly targeting microglia (the brain cells expressing the highest levels of its main receptor, CCR3^27^). Moreover, our data suggest that the preservation of cognitive function following heterochronic BMT may be mediated in part by reduced CCL11-mediated microglial activation, which would be expected to restrict synapse loss. The lack of an effect of heterochronic BMT on β2-microglobulin levels suggests that non-hematopoietic cells are the main source of this factor in old mice, and that while β2-microglobulin levels may increase with age and suppress neurogenesis^10^, reducing β2-microglobulin is not critical for preservation of cognitive function.

We chose to focus on CCL11 and β2-microglobulin because parabiosis studies have implicated these factors in aging-associated cognitive decline^3,10^. However, other circulating factors, including cytokines/chemokines, are also likely to operate in concert with CCL11. For instance, aging-associated increases in the production of several inflammatory mediators have been reported (‘inflammaging’, reviewed in ref. ^36^), both in the circulation and in the brain itself, and changes in circulating levels of several other chemokines have been correlated in a comparison of aging and heterochronic parabiosis^3^. Of particular relevance to the current study, CCL11 production may be a consequence of a shift in the T cell cytokine balance or elevated TNF-α production^34,37,38^. Hippocampal function may be impacted by signals derived from both myeloid and lymphoid cells in the periphery, and changes in immune cell composition, polarization and function may underlie the observed effects.

In future studies, it will also be interesting to determine whether transplantation of old bone marrow into young mice has detrimental effects on cognitive function i.e., increased glial activation and synapse loss, accompanied by reduced neurogenesis (which is more important in young mice), resulting in premature cognitive decline. Such studies will likely need to be performed with head-shielding to protect neural progenitors from the toxic effects of irradiation. However, our data provide additional support for the current clinical practice of using young donors for BMT in humans^39^.

Our study also demonstrates that restoration of neurogenesis is not essential for the preservation of cognitive function, and that microglial rejuvenation via peripheral manipulation of the hematopoietic system may be sufficient to maintain or restore hippocampal function. Moreover, our study presents a potential translational approach for treating neurological diseases associated with aging. Weekly transfusions of young plasma are already being evaluated in Alzheimer’s disease patients (The PLASMA Study, ClinicalTrial.gov NCT02256306)^40^, but heterochronic bone marrow reconstitution to replace old hematopoietic stem cells with young ones could provide therapeutic benefit for longer periods. Heterochronic BMT using traditional protocols may not currently be a feasible approach for preserving cognitive function in humans because of the risks associated with the transplantation procedure (availability of a compatible donor and the transient increased susceptibility to infection). However, in the future it may be possible to mitigate these risks by generating ‘personalized’ young hematopoietic stem cells from a patient’s own induced pluripotent stem cells^41^ (which would be rejuvenated during reprogramming), and delivering them in large numbers to competitively replace at least some of a patient’s endogenous hematopoietic stem cells. Based on our data, this rejuvenation technique could have long-term positive effects on aging, and perhaps slow the progression of aging-associated diseases such as Alzheimer’s and Parkinson’s disease.

## Methods

### Mice, bone marrow transplantation (BMT), and CCL11 injections

Male wild type (CD45.1 and CD45.2 congenic strains) and green fluorescent protein (GFP) transgenic mice (all on a C57BL/6 background) were purchased from The Jackson Laboratory (Bar Harbor, ME) and maintained in house. Mice were purchased at 3 months of age or 12 months of age and aged in house until the appropriate age for experiments. All procedures were approved by Cedars-Sinai Medical Center’s Institutional Animal Care and Use Committee.

Recipient old (18-month; CD45.2) mice were irradiated (9 Gy split dose from a gamma source i.e., two 4.5 Gy doses, 2 h apart, without head-shielding), and 4 h later received 2 million young (4-month; CD45.1) or old (18-month; CD45.2, or CD45.1 if indicated) donor bone marrow cells (pooled from the femurs and tibias of donor mice) by intravenous injection. Irradiation delivered without head shielding prior to injection of donor bone marrow cells enabled us to exclude the impact of neurogenesis, because irradiation is known to inhibit the proliferation of neural progenitors^15,16^. For BrdU analysis, mice received 3 intraperitoneal injections of 4 mg 5-bromo-2’-deoxyuridine (BrdU) daily on 5 consecutive days and were euthanized 2 h after the final injection. For CCL11 injections, mice received 4 intraperitoneal injections of recombinant mouse CCL11 (10 μg per kg; R&D Systems, Minneapolis, MN) over the course of 10 days, consistent with a previously published protocol^3^.

### Behavioral testing

Behavioral testing was performed in a blinded manner, 6 months post-transplantation.

Open field testing: Mice were placed in an open-topped, clear Plexiglass chamber and left undisturbed for 60 min. Activity (includes horizontal locomotion and vertical rearing), speed and distance were tracked by disturbances in a grid of photobeams in the chamber, which were recorded using tracking software (Noldus, Leesburg, VA) and analyzed at 5-min intervals.

Spontaneous alternation (Y-maze) testing: Mice were placed in an opaque, Y-shaped maze (San Diego Instruments, San Diego, CA) and spontaneous alternation of the mice between the three arms of the maze was recorded for 8 min.

Context- and cue-dependent fear conditioning: Fear-conditioning tests were performed as described previously^3^. Mice were placed in a chamber where they received a loud tone paired with a mild (0.3 mA) electric shock. To assess context-dependent fear conditioning, mice were placed in the same chamber the following day and freezing (indicative of fear of another shock) was recorded for 5 min. The following day, cue-dependent fear conditioning was assessed by placing the mice in a novel chamber where the tone was re-played and freezing was recorded for 5 min. Freezing was measured using the Freeze Monitor tracking system and software (San Diego Instruments, San Diego, CA) and is presented as the proportion of the evaluation period that mice exhibited freezing.

Barnes maze: Barnes maze testing was performed as described previously^18^. Briefly, mice were placed under a bright light on a circular platform with 20 holes around the perimeter, one of which provided access to a small dark chamber (escape box). Colored shapes were placed around the maze to serve as visual cues. Mice were trained to find the escape box 3 times per day for 4 days (training phase, days 1–4). After a 2-day break, mice were tested on the maze (day 7) and the number of errors and time to find the escape box were recorded. The following day, the escape box was moved to the opposite side of the maze (reversal phase) and the mice were tested again on 2 consecutive days (days 8–9).

### Histological analyses

Samples for histological analysis were randomly selected from independent experiments and pooled for analysis to control for potential differences between experiments. Histological assessments were performed in a blinded manner. Hemibrains were fixed in 4% paraformaldehyde (Electron Microscopy Sciences, Hatfield, PA) for 24 h and cryoprotected in 30% sucrose for 48 h. 30 μm coronal or sagittal sections were cut on a Leica SM2010R freezing sliding microtome and stored in Section Storage Media (30% Sucrose + 30% Ethylene Glycol in 0.1 M Phosphate Buffer) at 4 °C until used for immunohistochemistry.

1/12th of the brain of each mouse (8–10 sections) was stained for NeuN, Doublecortin (DCX), Iba1, LAMP1, CD68 and GFAP, and 1/24th of the brain (4–5 sections) was stained for CD11b and GFP. Sections were washed 3 times in Phosphate Buffered Saline (PBS) for 5 min and then blocked in 5% goat or donkey serum + 0.3% Triton X-100 for 1 h at room temperature. Primary antibodies were diluted in 5% serum + 0.3% Triton X-100 at the appropriate concentrations: NeuN (1:1000, Catalog # mab377, EMD Millipore, Temecula, CA), Doublecortin C-18 (DCX 1:500, Catalog # SC-8066, Santa Cruz Biotechnology Inc, Dallas, TX), 5-bromo-2’-deoxyuridine (BrdU 1:1000, Catalog # ab6326, Abcam, Cambridge, MA), GFAP (1:1000, Catalog # AB5804, EMD Millipore), Iba1 (1:1000, Catalog # 019-1974, Wako, Richmond, VA), LAMP1 (1:200, Catalog # SC-19992, Santa Cruz Biotechnology Inc, Dallas, TX), CD68 (1:200, Catalog # MCA1957, Bio-Rad, Hercules, CA), CD11b (1:500, Catalog # AB133357, Abcam, Cambridge, UK), GFP (1:1000, Catalog #AB5450, Abcam, Cambridge, UK) and incubated overnight at 4 °C. The following day, sections were washed 3 times (in PBS for 5 min) and then incubated with Alexa Fluor-conjugated antibodies (594 or 488, Thermo-Fisher Scientific, Waltham, MA) diluted 1:500 in 5% serum + 0.3% Triton X-100 for 1 h at room temperature. Sections were then washed, mounted and stained with DAPI (1:10,000, Catalog # D3571, Invitrogen, Waltham, MA).

For Homer1, VGlut1 and C3 immunostaining, 1/24th of the brain (4 sections) was stained as above, but sections were blocked in 20% goat or donkey serum + 0.3% Triton X-100 for 2 h at room temperature. Primary antibodies were diluted in 10% serum (or 5% bovine serum albumin) + 0.03% Triton X-100 at the appropriate concentrations: VGlut1 (1:1000, Catalog # AB5905, Millipore, Billerica, MA), Homer1 (1:200, Catalog # 160003, Synaptic Systems, Goettingen, Germany), and C3 (1:200, Catalog # 55713, MPBio Cappel, Santa Ana, CA), and incubated for 2 days at 4 °C. Alexa Fluor-conjugated secondary antibodies (Thermo Fisher Scientific, Waltham, MA) were diluted in 10% serum + 0.03% Triton X-100 at a concentration of 1:200 for 2 h at room temperature. Imaging was performed on a Nikon Eclipse Ti confocal microscope using a 60x oil objective with 2x averaging at a resolution of 2048 × 2048 and 24 frames per second.

### Quantification of histological data

NeuN and hippocampal volume: The total number of neurons in the CA1 and CA3 regions of the hippocampus was estimated using stereological counts (Stereo Investigator, MBF Bioscience, Williston, VT) of 1/12th of the brain (8–10 30 μm sections, about 400 μm apart) on a Leica DM600B microscope. The CA1 and CA3 regions were contoured at 5× and counted at 40× using the Optical Fractionator probe, with a counting frame of 50 × 50 μm and a grid size of 150 × 150 μm. The same tissue sections were then used to estimate the volume using the Cavalieri probe in StereoInvestigator. Briefly, the entire hippocampus was contoured for each section. A spacing of 0.01 mm^2^ was chosen for the point probe and the maximal number of points within the hippocampus was quantified and area was calculated from this information. Hippocampal volume was then estimated by multiplying area by section interval and thickness.

Doublecortin (DCX)^*+*^ cells: Stereological counts of brain sections were quantified as described previously^3^. The total number of DCX^+^ cells in each brain was estimated by counting the number of DCX^+^ cells in the dentate gyrus in every sixth hemibrain section and multiplying by twelve.

BrdU^*+*^ cells: BrdU incorporation was assessed in the dentate gyrus. 7–19 sections per mouse were stained and the mean BrdU^+^ cell number per section was quantified using a ×40 oil objective.

Homer1 and VGlut1 or C3 co-localization: The Spot Detection measurement tool (NIS Elements, Nikon Instruments, Melville, NY) was used to quantify synaptic puncta and co-localization. Numbers of puncta were determined by creating binary layers for each channel. To quantify co-localized puncta, an intersection layer of the two binary layers was generated and the number of intersecting puncta was quantified.

Spine density analysis: Golgi staining was performed on fresh hippocampal tissue using the FD Rapid Golgi Staining Kit (FD Neurotechnologies, Columbia, MD) according to the manufacturer’s directions. Images were obtained on a Nikon Eclipse Ti confocal microscope using a ×60 water objective and a transmitted detector. For each mouse, a combination of secondary and tertiary spines of 5 neurons in the CA3 region were imaged. Spine number and morphology were quantified using RECONSTRUCT software as described^42^.

GFAP^*+*^ cells: 8 images of the hippocampus were obtained per section (4 sections per mouse) at ×20 magnification using a Leica DM600B microscope. The CA1, CA3 and dentate gyrus regions of the hippocampus were represented. Total numbers of GFAP^+^ cells were quantified using ImageJ.

Astrocyte hypertrophy: Astrocyte morphology was assessed using Neurolucida (MBF Biosciences, Williston, VT). Astrocyte cell bodies were outlined in the following 3 regions of the hippocampus: CA1 (3 fields per section), CA3 (3 fields per section) and dentate gyrus (2 fields per section) across 2 sections per mouse. The perimeter and area of an average of ~100 astrocytes per mouse were quantified.

Iba1^*+*^, CD68^*+*^, and LAMP1^*+*^ cells: For each stain, 4 sections per mouse were quantified using ImageJ. For each section, one image was taken in each of the following 3 regions of the hippocampus: CA1, CA3 and dentate gyrus. Total numbers of microglia were quantified by counting all Iba-1^+^ cells that showed a complete cell body. CD68 and LAMP1 were quantified by counting positive-staining of Iba1^+^ cell bodies.

Microglial morphology: Morphology was assessed using Neurolucida (MBF Biosciences, Williston, VT). Z-stack images of Iba1^+^ sections were used to trace cell bodies and dendrites of 6–7 microglia per stack (2 stacks per mouse). The Branch Structure Analysis tool was used to quantify process complexity and average dendritic length.

### Real-time RT-PCR analysis

Samples were randomly selected from independent experiments and pooled for analysis to control for potential differences between experiments. Samples from each mouse were assessed in duplicate. RNA was extracted from fast-frozen hippocampi using Trizol (Thermo-Fisher Scientific, Waltham, MA) and an RNeasy Kit (Qiagen, Valencia, CA), and then digested using DNase I (RQ1 DNase, Promega, Madison, WI) according to the manufacturer’s protocol. Complementary DNA was generated from 0.5 μg total RNA using a reverse transcriptase kit (Thermo-Fisher Scientific, Waltham, MA) on a C1000 Thermal Cycler. Quantitative PCR analyses were run on a CFX384 Real-Time System with the SYBR Select Master Mix (Applied Biosystems, Foster City, CA). The primer pairs used are shown in Table 1.

**Table 1:** Real-time RT-PCR primers

Gene	Primer sequence
*C1qa*	Forward: 5′ GAA AGG CAA TCC AGG CAA TA 3′
	Reverse: 5′ CTG GTT GGT GAG GAC CTT GT 3′
*C3*	Forward: 5′ AAG CAT CAA CAC ACC CAA CA 3′
	Reverse: 5′ CTT GAG CTC CAT TCG TGA CA 3′
*Gfap*	Forward: 5′ ACA AGG ACG TGG TGA TGT GA 3′
	Reverse: 5′ CAG AAG GAA GGG AAG TGC TG 3′
*Iba1*	Forward: 5′ TGA GGA GCC ATG AGC CAA AG 3′
	Reverse: 5′ GCT TCA AGT TTG GAC GGC AG 3′
*Cd68*	Forward: 5′ GGG GCT CTT CGG AAC TAC AC 3′
	Reverse: 5′ GTA CCG TCA CAA CCT CCC TG 3′
*Gapdh*	Forward: 5′ GTG TTC CTA CCC CCA ATG TGT 3′
	Reverse: 5′ ATT GTC ATA CCA GGA AAT GAG CTT 3′

### Plasma collection and ELISA analysis

Mouse blood was collected into EDTA-coated tubes via mandibular vein or intracardial bleeding. Tubes were centrifuged and plasma was collected and stored at −80 °C. ELISA assessments of CCL11 (R&D Systems, Minneapolis, MN) and β2-microglobulin (Lifespan BioSciences, Inc., LS-F14141, Seattle, WA) levels in the plasma samples (in triplicate) were performed according to the manufacturers’ directions.

### Statistical analyses

GraphPad Prism versions 6 and 7 were used for statistical analyses. Student’s *t*-test (CCL11 injection experiments), repeated measures ANOVA (lymphocyte counts and Barnes Maze testing) and one-way ANOVA (all other experiments) were used to evaluate statistically significant differences between groups. For all ANOVA testing, Tukey-Kramer post-hoc analysis was performed to correct for multiple comparisons. All testing was performed at the two-sided alpha level of 0.05. Multi-way ANOVA was used to justify the pooling of data from 2–3 independent experiments for open field, spontaneous alternation (Y-maze) and Barnes maze testing.

### Reporting summary

Further information on experimental design is available in the Nature Research Reporting Summary linked to this article.

## Supplementary information

Supplementary Information

Supplementary Data 1

Description of Additional Supplementary Files

Reporting Summary

## Data Availability

The authors declare that the data supporting the findings of this study are available within the paper and its supplementary files (including all raw data in the Supplementary Data 1).
