# Supplementary material for: Young bone marrow transplantation preserves learning and memory in old mice
Source: Commun Biol. 2019 Feb 20;2:73. doi: 10.1038/s42003-019-0298-5 (PMC6382867; doi:10.1038/s42003-019-0298-5)
Supplement: Supplementary file 1 — Supplementary Information [file 42003_2019_298_MOESM1_ESM.pdf]

Supplementary Figure 1

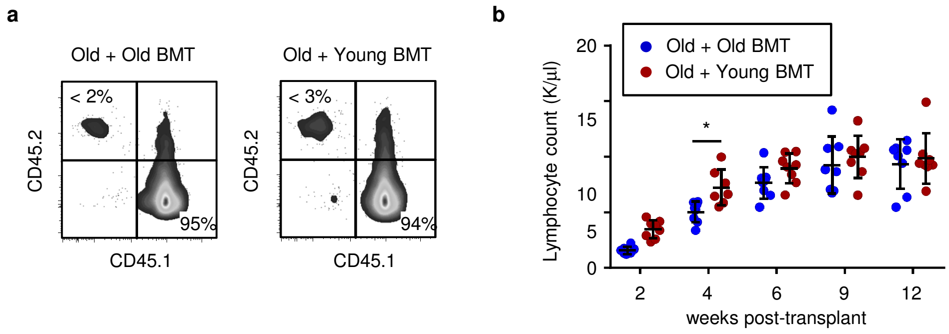

Supplementary Figure 1. Transplantation of young and old bone marrow into old mice. (a, b) Hematopoietic reconstitution following transplantation of CD45.1$^+$ donor bone marrow cells into CD45.2$^+$ recipient mice was assessed 6 months post-transplantation (a; representative plots are shown) or at the indicated time points (b). (a) CD45.1 and CD45.2 expression by white blood cells (enriched from total blood cells by red blood cell lysis) was assessed by flow cytometry. Data shown are representative of >20 transplanted mice. (b) Circulating lymphocytes were assessed by complete blood count (CBC) analysis at the indicated timepoints post-transplantation (n = 8 mice per group). The dotplot shows mean plus standard deviation. *p<0.05 (ANOVA)

Supplementary Figure 2

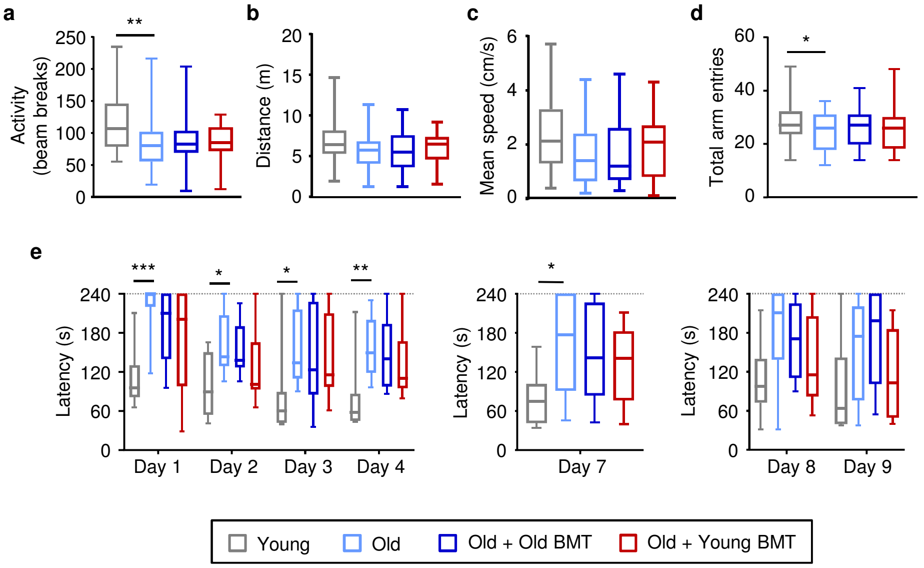

Supplementary Figure 2. Additional analyses of cognitive function following bone marrow transplantation. 18-month old mice received a bone marrow transplant from either young (4-month) or old (18-month) donors. 6 months post-transplantation, the cognitive function of recipient mice, as well as non-transplanted old (24-month) and young (4-month) control mice, was evaluated. (a-c) Activity (a; includes horizontal locomotion and vertical rearing), distance covered (b), and mean speed (c) were assessed during the last 5 minutes of a 1-hour period in an open field test. n = 32-51 mice per group, pooled from 3 independent experiments. Justification for pooling was confirmed by multi-ANOVA analysis. (d) The total number of arm entries made in a Y-maze was assessed over an 8-min period. n = 44-46 mice per group, pooled from 3 independent experiments. Justification for pooling was confirmed by multi-ANOVA analysis. Similarly significant differences or trends towards significance were observed in all independent experiments. (e) Learning and memory were assessed in a Barnes Maze test. Mice were evaluated for their ability to recall the location of an escape hole. After the learning phase (days 1-4), mice were rested for 2 days and then memory was evaluated (day 7). The position of the escape hole was then changed, and the ability of the mice to discover and remember the new location was re-evaluated (days 8-9). The time taken to successfully locate the escape hole (latency) was recorded up to a maximum of 240 seconds. n = 10-15 mice per group. Box and whisker plots show median, 25th and 7th percentile, maximum and minimum values. *p<0.05, **p<0.01, ***p<0.001 (ANOVA with Tukey-Kramer post-hoc test)

Supplementary Figure 3

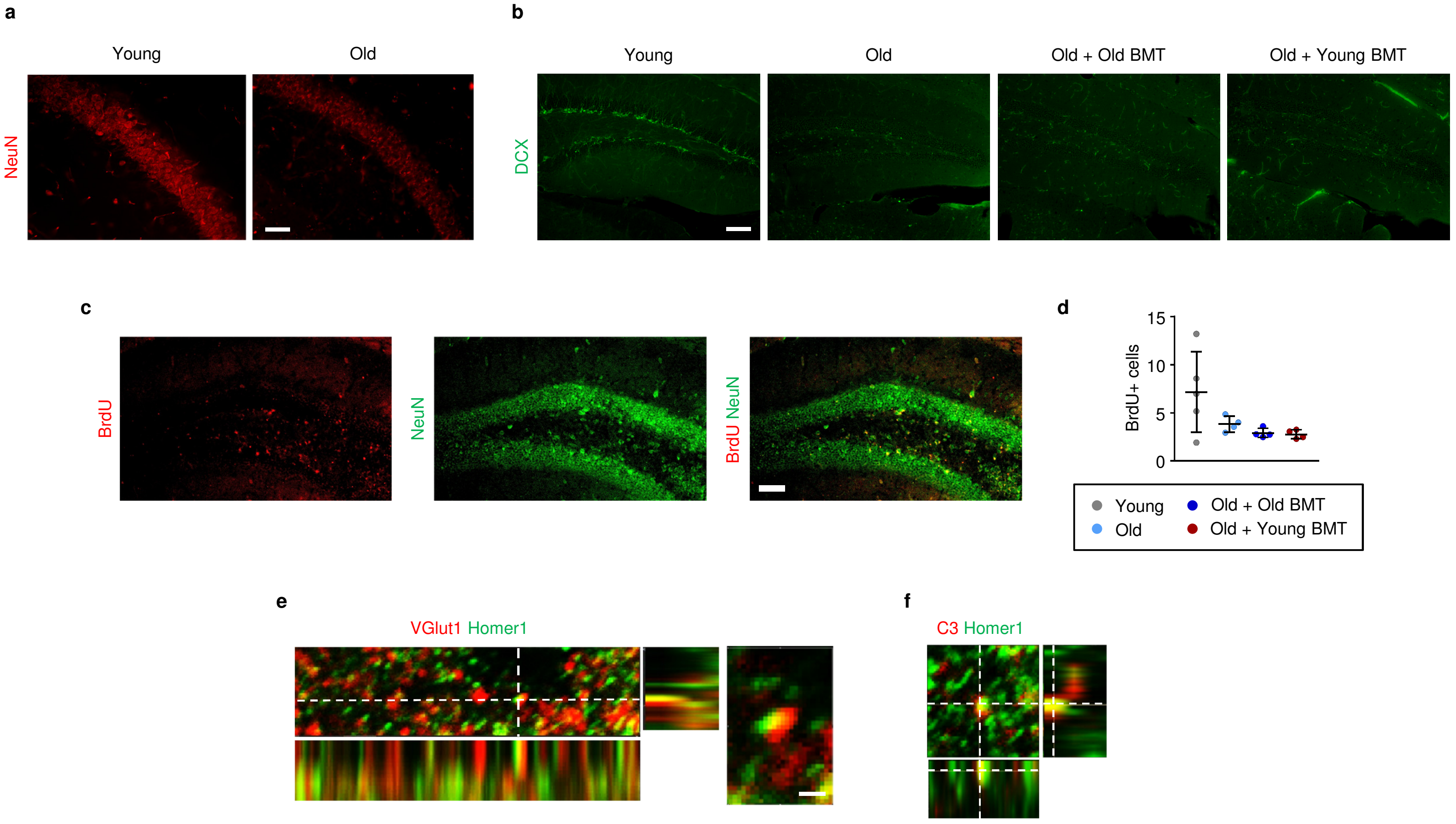

Supplementary Figure 3. Analysis of hippocampal neurons following bone marrow transplantation. 18-month old mice received a bone marrow transplant from either young (4-month) or old (18-month) donors, and were euthanized 6 months (a-b, e-f) or 1 month (c-d) post-transplant. (a-b) Representative images of NeuN staining (a; scale bar 50 µm) and DCX staining (b; scale bar 100 µm). (c-d) BrdU incorporation was assessed in the dentate gyrus 1 month post-transplant. A representative image is shown (c; scale bar 75 µm) and $BrdU^{+}$ cells were quantified (d). n = 4-5 mice per group from 1 experiment. (e-f) Representative images of co-localized synaptic markers VGlut1 and Homer1 (e; scale bar 2 µm) and C3 deposition on synapses (f) are shown.

Supplementary Figure 4

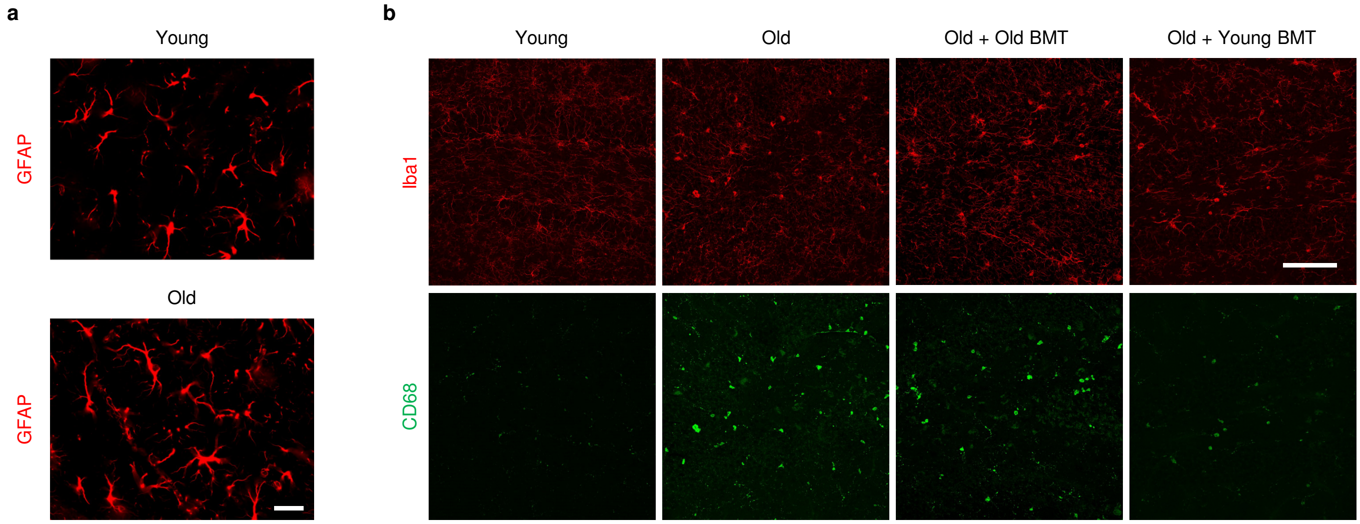

Supplementary Figure 4. Analysis of glial cell activation in the hippocampus following bone marrow transplantation. 18-month old mice received a bone marrow transplant from either young (4-month) or old (18-month) donors, and were euthanized 6 months post-transplant. Representative images of GFAP staining (a; scale bar 25 µm), and Iba1 and CD68 staining (b; scale bar 50 µm) are shown.

Supplementary Figure 5

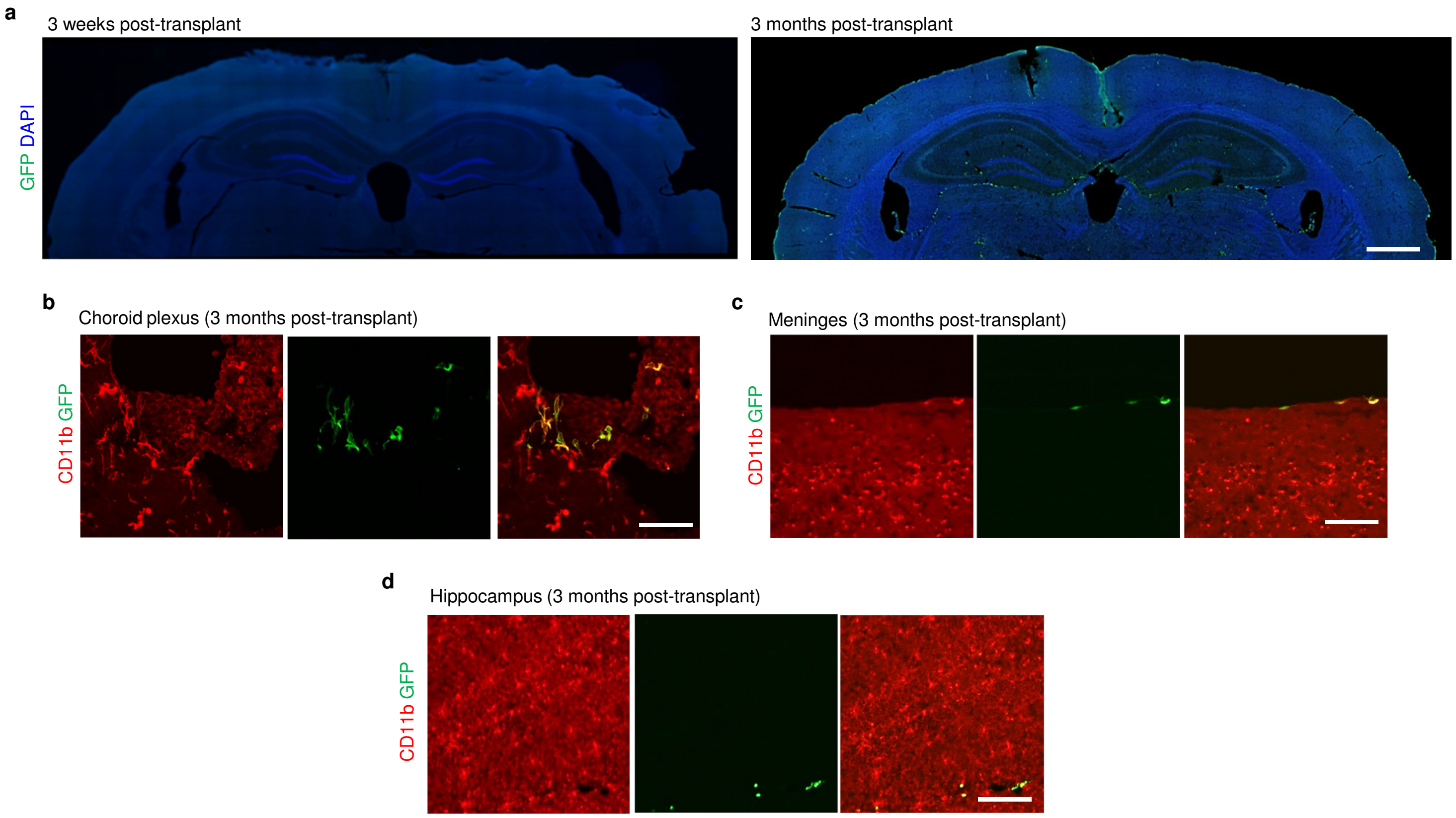

Supplementary Figure 5. Evaluation of donor-derived cells in the brain following bone marrow transplantation. Old (18-month) mice received a bone marrow transplant from young (4-month) GFP transgenic donor mice, and the presence of GFP^{+} donor-derived cells in the brains of recipient mice was evaluated 3 months post-transplantation. Images are representative of 12 recipient mice. Scale bars: 500 µm in (a), 5 µm in (b-d).

Supplementary Figure 6

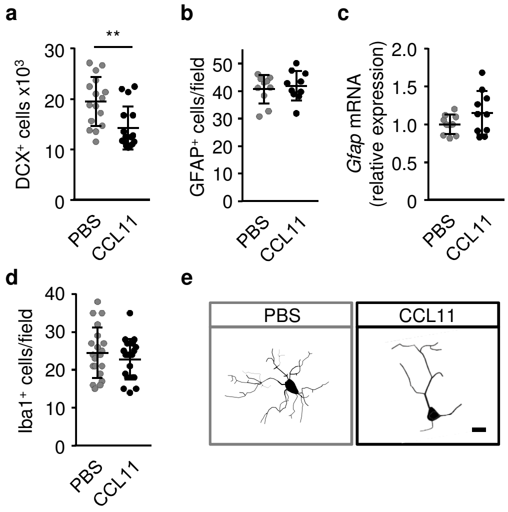

Supplementary Figure 6. Evaluation of neurons, astrocytes and microglia following CCL11 injection.

CCL11 was administered to young (4-month) mice by intraperitoneal injection 4 times over a period of 10 days. Histological and molecular analyses of the hippocampus were then performed. (a, b) DCX^+ cells (a; n = 16 mice per group, pooled from 2 independent experiments) and $GFAP^+$ cells (b; n = 10 mice per group, pooled from 2 independent experiments) were evaluated by immunostaining. (c) *Gfap* mRNA expression was assessed by RT-PCR (normalized to young non-transplanted mRNA; n = 5 mice per group). (d) $Iba1^+$ cells were evaluated by immunostaining (n = 10 mice per group, pooled from 2 independent experiments). (e) Representative images of microglial morphology (scale bar 5 μm). The dotplots show mean plus standard deviation. **p<0.01 (Student's t-test)
