# Supplementary material for: Young bone marrow transplantation preserves learning and memory in old mice
Source: Commun Biol. 2019 Feb 20;2:73. doi: 10.1038/s42003-019-0298-5 (PMC6382867; doi:10.1038/s42003-019-0298-5)
Supplement: Supplementary file 3 — Description of Additional Supplementary Files [file 42003_2019_298_MOESM3_ESM.docx]

**Description of Additional Supplementary Files**

**File Name**: Supplementary Data 1

**Description**: This file contains all data presented in the main and supplementary figures.
