# Supplementary material for: Young bone marrow transplantation preserves learning and memory in old mice
Source: Commun Biol. 2019 Feb 20;2:73. doi: 10.1038/s42003-019-0298-5 (PMC6382867; doi:10.1038/s42003-019-0298-5)
Supplement: Supplementary file 4 — Reporting Summary [file 42003_2019_298_MOESM4_ESM.pdf]

nature research

Corresponding author(s): Helen Goodridge, Clive Svendsen

☐ Initial submission ☐ Revised version ☒ Final submission

Life Sciences Reporting Summary

Nature Research wishes to improve the reproducibility of the work that we publish. This form is intended for publication with all accepted life science papers and provides structure for consistency and transparency in reporting. Every life science submission will use this form; some list items might not apply to an individual manuscript, but all fields must be completed for clarity.

For further information on the points included in this form, see Reporting Life Sciences Research. For further information on Nature Research policies, including our data availability policy, see Authors & Referees and the Editorial Policy Checklist.

▸ Experimental design

1. Sample size

Describe how sample size was determined.	Sample sizes were chosen based on pilot experiments in order to provide sufficient power for statistical comparison. Power calculations were performed using sample size calculator software available on the internet with alpha = 0.05 and power = 0.8.

2. Data exclusions

Describe any data exclusions.	No data were excluded from analysis.

3. Replication

Describe whether the experimental findings were reliably reproduced.	Bone marrow transplantation was performed in 4 independent experiments, and CCL11 injection was performed in 2 independent experiments. Behavioral analyses were each performed in 2-3 experiments, and histological and molecular analyses were performed using samples randomly selected from 2-3 experiments. Data presented in the manuscript were reproducible, and replicates for each experiment are described in the figure legends.

4. Randomization

Describe how samples/organisms/participants were allocated into experimental groups.	Mice were randomly allocated to treatment groups prior to any experimental manipulations. Samples were randomly selected from multiple experiments for histological and molecular analyses.

5. Blinding

Describe whether the investigators were blinded to group allocation during data collection and/or analysis.	Investigators were blinded to animal treatment groups during behavioral testing.<br><br>All immunohistochemistry, RNA and ELISA experiments were performed by a blinded investigator and unblinded after completion of data collection.<br><br>Finally, during data analysis, each group was blinded for the investigator and labeled as "Group A", "Group B" etc. Experimental treatments for each group were revealed after data were analyzed.

Note: all studies involving animals and/or human research participants must disclose whether blinding and randomization were used.

6. Statistical parameters

For all figures and tables that use statistical methods, confirm that the following items are present in relevant figure legends (or in the Methods section if additional space is needed).

n/a	Confirmed	
☐	☒	The exact sample size (*n*) for each experimental group/condition, given as a discrete number and unit of measurement (animals, litters, cultures, etc.)
☐	☒	A description of how samples were collected, noting whether measurements were taken from distinct samples or whether the same sample was measured repeatedly
☐	☒	A statement indicating how many times each experiment was replicated
☐	☒	The statistical test(s) used and whether they are one- or two-sided (note: only common tests should be described solely by name; more complex techniques should be described in the Methods section)
☐	☒	A description of any assumptions or corrections, such as an adjustment for multiple comparisons
☐	☒	The test results (e.g. *P* values) given as exact values whenever possible and with confidence intervals noted
☐	☒	A clear description of statistics including central tendency (e.g. median, mean) and variation (e.g. standard deviation, interquartile range)
☐	☒	Clearly defined error bars

See the web collection on statistics for biologists for further resources and guidance.

Software

Policy information about availability of computer code

7. Software

Describe the software used to analyze the data in this study.	GraphPad Prism versions 6 and 7 were used for statistical analyses in this study.

For manuscripts utilizing custom algorithms or software that are central to the paper but not yet described in the published literature, software must be made available to editors and reviewers upon request. We strongly encourage code deposition in a community repository (e.g. GitHub). *Nature Methods* guidance for providing algorithms and software for publication provides further information on this topic.

Materials and reagents

Policy information about availability of materials

8. Materials availability

Indicate whether there are restrictions on availability of unique materials or if these materials are only available for distribution by a for-profit company.	No unique materials were used.

9. Antibodies

Describe the antibodies used and how they were validated for use in the system under study (i.e. assay and species).

NeuN: EMD Millipore, Temecula CA, Catalog: MAB377, Clone: A60
Validation: Clone and lot was validated for use in mouse brain tissue according to the manufacturer's website: http://www.emdmillipore.com/US/en/product/Anti-NeuN-Antibody%2C-clone-A60,MM_NF-MAB377

Doublecortin: Santa Cruz Biotechnology, Dallas TX, Catalog: SC-8066, Clone: C-18
Validation: Clone and lot was validated for use in mouse brain tissue according to the manufacturer's website: https://www.scbt.com/scbt/product/doublecortin-antibody-c-18
N.B. this antibody clone has been discontinued.

Iba1: Wako, Richmond VA, Catalog: 019-1974
Validation: Antibody was validated for use in mouse brain tissue according to the manufacturer's website: http://www.wako-chem.co.jp/english/labchem/product/life/AntiIba1/index.htm#02

LAMP1: Santa Cruz Biotechnology, Dallas TX, Catalog: SC-19992, Clone: 1D4B
Validation: Lot was validated for use in mouse brain tissue according to the manufacturer's website: https://www.scbt.com/scbt/product/lamp-1-antibody-1d4b

CD68: Bio-Rad, Hercules CA, Catalog: MCA1957, Clone: FA-11
Validation: Antibody was validated for use in mouse tissue according to the manufacturer's website: https://www.bio-rad-antibodies.com/mouse-cd68-antibody-fa-11-mca1957.html

GFAP: EMD Millipore, Temecula CA, Catalog: AB5804
Validation: Antibody was validated for use in mouse tissue according to the manufacturer's website: http://www.emdmillipore.com/US/en/product/Anti-Glial-Fibrillary-Acidic-Protein-%28GFAP%29-Antibody,MM_NF-AB5804

CD11b: Abcam, Cambridge MA, Catalog: AB133357
Validation: Antibody was validated for use in mouse tissue according to the manufacturer's website: http://www.abcam.com/cd11b-antibody-epr1344-ab133357.html

GFP: Abcam, Cambridge MA, Catalog: AB5450
Validation: Antibody was validated according to the manufacturer's website: http://www.abcam.com/gfp-antibody-ab5450.html

Homer1: Synaptic Systems, Goettingen GE, Catalog: 160003
Validation: Antibody was validated for use in mouse tissue according to the manufacturer's website: https://www.sysy.com/products/homer1/facts-160003.php

VGlut1: EMD Millipore, Temecula CA, Catalog: AB5905
Validation: Antibody was validated for use in mouse brain tissue according to the manufacturer's website: https://www.emdmillipore.com/US/en/product/Anti-Vesicular-Glutamate-Transporter-1-Antibody,MM_NF-AB5905?ReferrerURL=https%3A%2F%2Fwww.google.com%2F

C3: MPBio Cappel, Santa Ana CA, Catalog: 0855713
Validation: Antibody was validated in brain tissue from C3 KO mice in the following study: Q. Shi et al., Complement C3-Deficient Mice Fail to Display Age-Related Hippocampal Decline. J Neurosci 35, 13029-13042 (2015).

BrdU: Abcam, Cambridge MA, Catalog: ab6326
Validation: Antibody was validated according to the manufacturer's website: https://www.abcam.com/brdu-antibody-bu175-icr1-ab6326.html

10. Eukaryotic cell lines

a. State the source of each eukaryotic cell line used.	No eukaryotic cell lines were used in this study.
b. Describe the method of cell line authentication used.	No eukaryotic cell lines were used in this study.
c. Report whether the cell lines were tested for mycoplasma contamination.	No eukaryotic cell lines were used in this study.
d. If any of the cell lines used are listed in the database of commonly misidentified cell lines maintained by ICLAC, provide a scientific rationale for their use.	No commonly misidentified cell lines were used in this study.

Animals and human research participants

Policy information about studies involving animals; when reporting animal research, follow the ARRIVE guidelines

11. Description of research animals

Provide details on animals and/or animal-derived materials used in the study.	Male wild type (CD45.1 and CD45.2 congenic strains) and green fluorescent protein (GFP) transgenic mice (all on a C57BL/6 background) were purchased from The Jackson Laboratory (Bar Harbor, ME). Mice were purchased at 3 months of age or 12 months of age and aged in-house until the appropriate age for experiments.

Policy information about studies involving human research participants

12. Description of human research participants

Describe the covariate-relevant population characteristics of the human research participants.	This study did not involve human research participants.
